# Differential immune cell infiltrations between healthy periodontal and chronic periodontitis tissues

**DOI:** 10.1186/s12903-020-01287-0

**Published:** 2020-10-27

**Authors:** Wei Li, Zheng Zhang, Zuo-min Wang

**Affiliations:** 1 Department of Stomatology, Beijing Chao-Yang Hospital, Capital Medical University, 8th Gongti South Road, Beijing, 100020 China; 2 Department of Periodontology, Tianjin Stomatological Hospital, Tianjin Key Laboratory of Oral Function Reconstruction, Hospital of Stomatology, Nankai University, 75th Dagu North Road, Tianjin, 300000 China

**Keywords:** Periodontitis, Host immunity, Inflammation, Infiltrating immune cells and subtypes

## Abstract

**Background:**

Host immunity plays an important role against oral microorganisms in periodontitis.

**Methods:**

This study assessed the infiltrating immune cell subtypes in 133 healthy periodontal and 210 chronic periodontitis tissues from Gene Expression Omnibus (GEO) datasets using the CIBERSORT gene signature files.

**Results:**

Plasma cells, naive B cells and neutrophils were all elevated in periodontitis tissues, when compared to those in healthy controls. In contrast, memory B cells, resting dendritic, mast cells and CD4 memory cells, as well as activated mast cells, M1 and M2 macrophages, and follicular helper T cells, were mainly present in healthy periodontal tissues. Furthermore, these periodontitis tissues generally contained a higher proportion of activated CD4 memory T cells, while the other subtypes of T cells, including resting CD4 memory T cells, CD8 T cells, follicular helper T cells (T_FH_) and regulatory T cells (Tregs), were relatively lower in periodontitis tissues, when compared to healthy tissues. The ratio of dendritic and mast cells and macrophages was lower in periodontitis tissues, when compared to healthy tissues. In addition, there was a significant negative association of plasma cells with most of the other immune cells, such as plasma cells *vs.* memory B cells (γ = − 0.84), plasma cells *vs.* resting dendritic cells (γ = − 0.64), plasma cells *vs.* resting CD4 memory T cells (γ = 0.50), plasma cells versus activated dendritic cells (γ = − 0.46), plasma cells versus T_FH_ (γ = − 0.46), plasma cells versus macrophage M2 cells (γ = − 0.43), or plasma cells versus macrophage M1 cells (γ = − 0.40), between healthy control and periodontitis tissues.

**Conclusion:**

Plasma cells, naive B cells and neutrophils were all elevated in periodontitis tissues. The infiltration of different immune cell subtypes in the periodontitis site could lead the host immunity against periodontitis.

## Background

Periodontitis is a chronic inflammatory disease in the surrounding tooth tissues [1] characterized by periodontal plaque microorganism-induced inflammation and loss of periodontal attachment [2]. This occurs in approximately 10–15% of the global population as the second main cause of tooth loss among adults [3, 4]. In the early stage of the disease, periodontal supporting tissues present as swollen, red, and/or bleeding (signs of acute inflammation), while in the later stage of the disease, the gums would draw back from the teeth, leading to jaw bone atrophy and tooth loss, and present with acute inflammation symptoms [2–4]. Plaque is the most important initial factor in periodontitis onset. However, plaque alone may not be enough to cause damage to the host periodontal tissue. This is caused by the host’s excessive immune response to plaque. Host immunity plays an important role against oral microorganisms, which is mainly through neutrophil, macrophage, and T and B-lymphocytes, in order to produce various cytokines and chemokines, and in turn, maintain the tissue homeostasis in the oral cavity [5–8]. To date, the precise molecular mechanisms of oral microorganism-induced disease or protection remains poorly understood [9]. For example, even single and low-abundance species of microorganisms can alter the host-microbial homeostasis to induce an inflammatory disease [10]. Furthermore, Curtis et al. reported that *Porphyromonas gingivalis* is a keystone pathogen of periodontitis [11]. The maintenance and restoration of oral tissue homeostasis after exposure to pathogens are essential to conquer oral inflammation, and the former depends on the complex coordination of innate and adaptive immune responses. In this regard, the evaluation and identification of tissue-specific immune cell types can help to illustrate the local immunoreactivity and severity of the inflammation. For example, a previous study investigated the development of chronic gingivitis. It was revealed that there was a decrease in fibroblasts (57–39%), and an increase in plasma cells (0.2–10.0%), while the portion of lymphocytes and macrophages remained stable [12]. To date, immunohistochemistry and flow cytometry are the common methodologies for the subtyping of immune cells in tissues, but these do possess some limitations [13]. Thus, the newly developed CIBERSORT technique would allow for the profiling and subtyping of immune cells in tissue specimens for gene expression profiles [13–15]. CIBERSORT is a method developed by Newman et al*.* [16] to analyze and characterize cell types in complex tissues using their gene expression profiles. Thus, in the present study, the investigators utilized the publically accessible Gene Expression Omnibus (GEO) web data, and applied this for the original CIBERSORT gene signature file [17–20], which profiled and analyzed the different immune cell subtypes between 133 healthy human periodontal tissues and 210 chronic periodontitis tissues. It is expected that the present study would provide useful information regarding the immune cell subpopulations in periodontitis, which could lead to the future control or prevention of periodontitis.

## Methods

### Database and data acquisition

In the present study, the investigators searched the GEO database (https://www.ncbi.nlm.nih.gov/geo/) using the following keywords: "periodontitis," "patient," "gingival tissues," and "gene expression". A total of four microarray datasets were obtained: GSE10334, GSE16134, GSE23586 and GSE54710 [17–20]. Then, the investigators retrieved the basic information of these datasets, but excluded two datasets (GSE23586 and GSE54710) (The reasons were because GSE54710 was a microRNA microarray dataset, which is not the focus of the study, and GSE23586 only contained a very small sample size [*n* = 6]). Thus, in the present study, the investigators included two datasets (GSE10334 and GSE16134) for the subsequent data analyses by downloading both GSE10334 [17] and GSE16134 [18] datasets from https://www.ncbi.nlm.nih.gov/geo/query/acc.cgi?acc=GSE10334 and https://www.ncbi.nlm.nih.gov/geo/query/acc.cgi?acc=GSE16134, respectively. Next, the investigators manually organized the expression profiles of each sample and the corresponding clinical data to obtain the data on the public available gene expression profiles. Each patient in the database contained 2–3 pieces of inflamed gingival tissues for periodontitis tissues (*n* = 210) and human healthy periodontal tissues (*n* = 133). The investigators took the average value of these multiple pieces of tissues for the data calculation and data analysis in the present study.

### Evaluation of infiltrating immune cells in periodontal tissues

Next, the investigators applied the CIBERSORT gene signature file LM22, which contained 22 subtypes of the infiltrating immune cell gene profile, according to a previous study [16], in order to profile and analyze the immune cell subtypes in 133 healthy human periodontal tissues and 210 chronic periodontitis tissues. Specifically, the differentially expressed gene datasets were analyzed with the standard annotation in the CIBERSORT website (https://cibersort.stanford.edu/) using the algorithm run with the default signature matrix at 1000 permutations. The reached CIBERSORT-derived *P*-value using the Monte Carlo sampling provided a confidence measure for each of the generated results.

### Statistical analysis

A total of 22 immune cell subtypes were identified and evaluated using the CIBERSORT metrics. Then, these were statistically analyzed using the Pearson correlation coefficient test. Each sample was quantified for the CIBERSORT-derived *P*-value and root mean squared error (RMSE). Afterwards, the profile of immune cells and the mean value for each tissue type were calculated for each sample, that is, the total macrophage proportion was divided into M0, M1 and M2, while T cells were classified into CD8+ T cells, CD4+ naive T cells, CD4+ memory resting T cells, CD4+ memory activated T cells, follicular helper T cells, regulatory T cells (Tregs), and T cells gamma delta. Student’s *t*-test was performed to analyze the difference between two types of tissue samples, while Pearson’s correlation coefficient test was used to analyze the other immune cell types. All statistical analyses were conducted using the SPSS 13.0 software (SPSS, Chicago, IL, USA).

## Results

### Baseline characteristics of the datasets

The present study obtained and analyzed the differentially expressed gene profiles between human healthy periodontal tissues (*n* = 133) and periodontitis tissues (*n* = 210), and applied the CIBERSORT gene signature file LM22 [16] to assess the different immune cell proportions in 133 healthy human periodontal tissues and 210 chronic periodontitis tissues. Briefly, these tissue samples were taken from 210 non-smokers with periodontitis, and each contributed to a ≥ 2 “diseased” interproximal papillae (with bleeding on probing [BoP], probing pocket depth [PPD] ≥ 4 mm, and clinical attachment loss [CAL] of ≥ 3 mm), or “healthy” papilla (no BoP; PPD of ≤ 4 mm and CAL of ≤ 2 mm), when such data was available (Table 1). However, merely the GSE10334 dataset provided the patient-specific clinicopathological parameters for a mean age of 42 years old, and 28 teeth, PD 3.9 mm, AL 4.1 mm and 71% BoP. Among these gingival tissues, 67% had a PD of ≥ 5 mm, while 62% had an AL of ≥ 5 mm [17].

**Table 1:** Characteristics of the individual GEO database data

GEO gene set ID	GSE10334	GSE16134
*Platform*	GPL570: Affymetrix Human Genome U133 plus 2.0 Array	
Number of subjects (control versus periodontitis)	64 versus 90	69 versus 120
*Clinical data*		
Healthy control	PD ≤ 4 mm, CAL ≤ 2 mm, BoP (−)	PD ≤ 4 mm, CAL ≤ 2 mm, BoP (−)
Chronic periodontitis	PD > 4 mm, CAL ≥ 3 mm, BoP ( +)	PD > 4 mm, CAL ≥ 3 mm, BoP ( +)
% of sites with bleeding on probing	71 ± 0.2	Unknown
Pocket depth (PD; mm)	3.9 ± 0.7	Unknown
Clinical attachment level (CAL; mm)	4.1 ± 0.9	Unknown
Tissue samples in specified PD range (mm, %)		Unknown
1–2	19	
3–4	14	
5	31	
≥ 6	36	
Diabetes	Not	Not
Smoking	Not	Not
	No systemic antibiotics or anti-inflammatory drugs for ≥ 6 months	No systemic antibiotics or anti-inflammatory drugs for ≥ 6 months
PubMed ID	18,980,520	19,835,625

*BoP* bleeding on probing, *CAL* clinical attachment loss, *PPD* probing pocket depth

### Distribution of tissue-infiltrated immune cell subtypes in the two databases

**Fig. 1:**
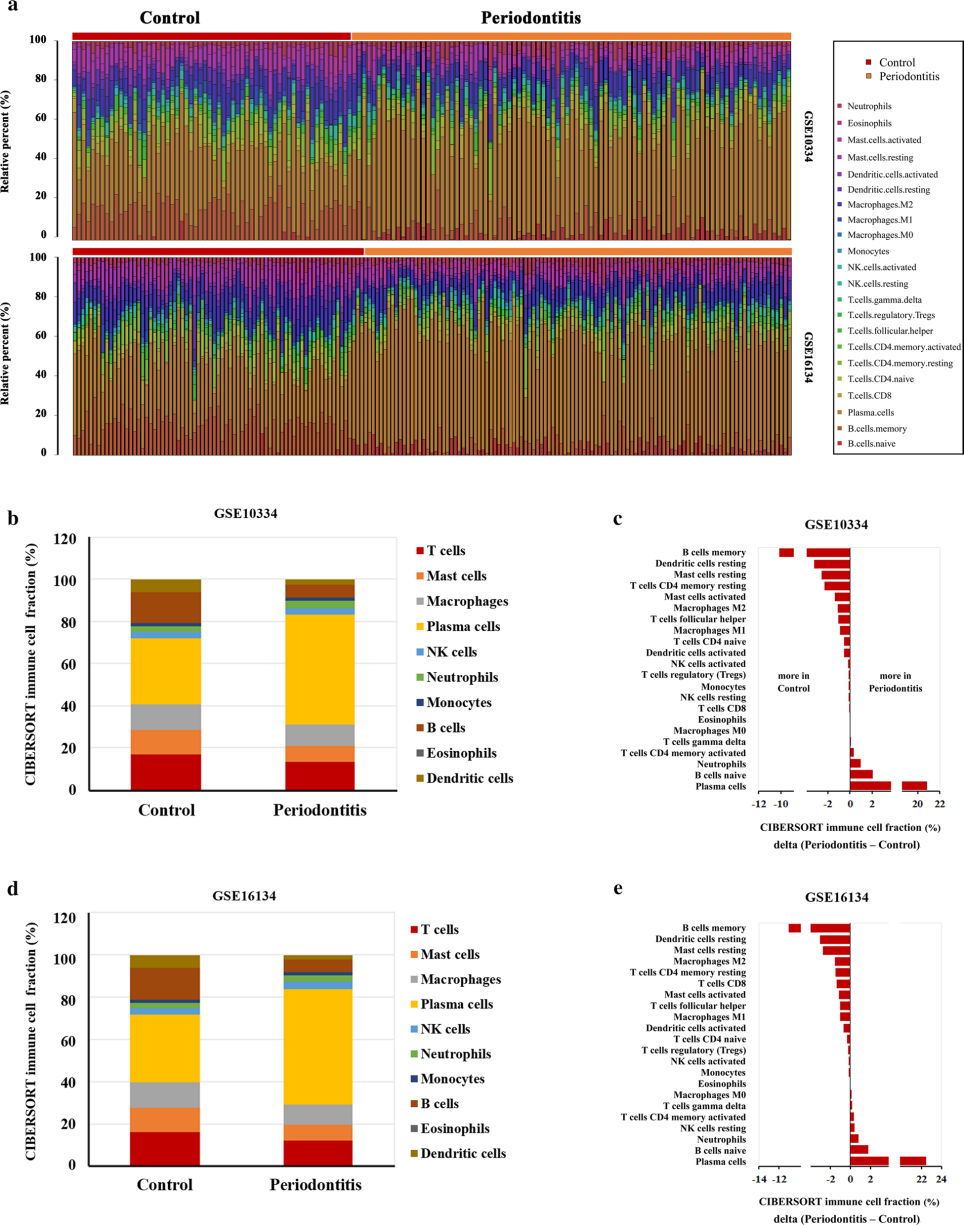
Differential level of immune cells in each healthy and chronic periodontitis sample. **a** The different colors and bar lengths indicate the levels of immune cell populations from the two databases. **b**–**e** The immune cell composition in the chronic periodontitis and control group in these two databases

Next, the investigators initially compared the data between these two datasets, and found that the composition ratio of the immune cell subtypes in the periodontitis or control group was similar (Fig. 1a, b and d). Specifically, the plasma and naive B cells and neutrophils were all elevated in the periodontitis group, when compared to those for healthy controls (Fig. 1), while memory B cells, resting dendritic, mast and CD4 memory cells, as well as activated mast cells, M1 and M2 macrophages, and follicular helper T cells, were higher in healthy periodontal tissues versus periodontitis tissues (Fig. 1c and e).

### The changes in immune cells subtypes in healthy and inflammatory periodontal tissues after merging the two databases

**Fig. 2:**
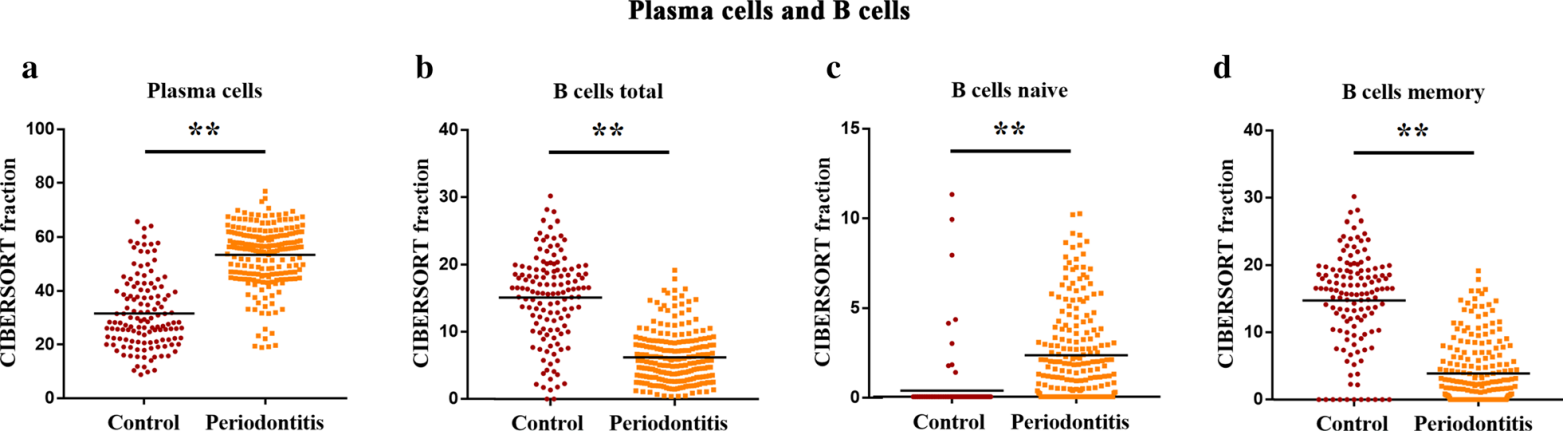
Differential level of adaptive immunity cells in human healthy and periodontitis tissues. The CIBERSORT immune cell fractions were assessed for each patient, and each dot represents a patient. The mean values and standard deviations of each cell subset for each patient group were identified for plasma cells (**a**), total B cells (**b**), naive B cells (**c**), and memory B cells (**d**). **P < 0.01, using Student’s *t*-test

**Fig. 3:**
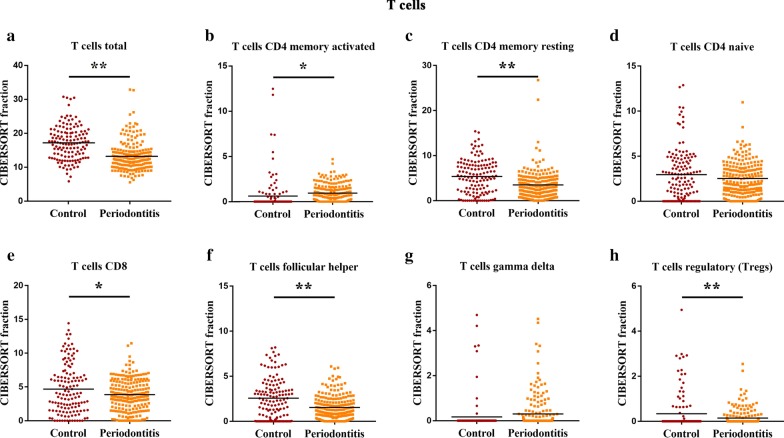
The differential level of T cell subtypes between human healthy and periodontitis tissues. The CIBERSORT immune cell fractions were determined for each patient, and each dot represents a patient. The mean values and standard deviations for each cell subset for each patient group were identified for the total T cells (**a**), CD4 memory activated cells (**b**), CD4 memory resting cells (**c**), CD4 naive cells (**d**), CD8 cells (**e**), follicular helper T cells (TFH) (**f**), T cells gamma delta (**g**) and Tregs (**h**). *P < 0.05 and **P < 0.01, using Student’s *t*-test

**Fig. 4:**
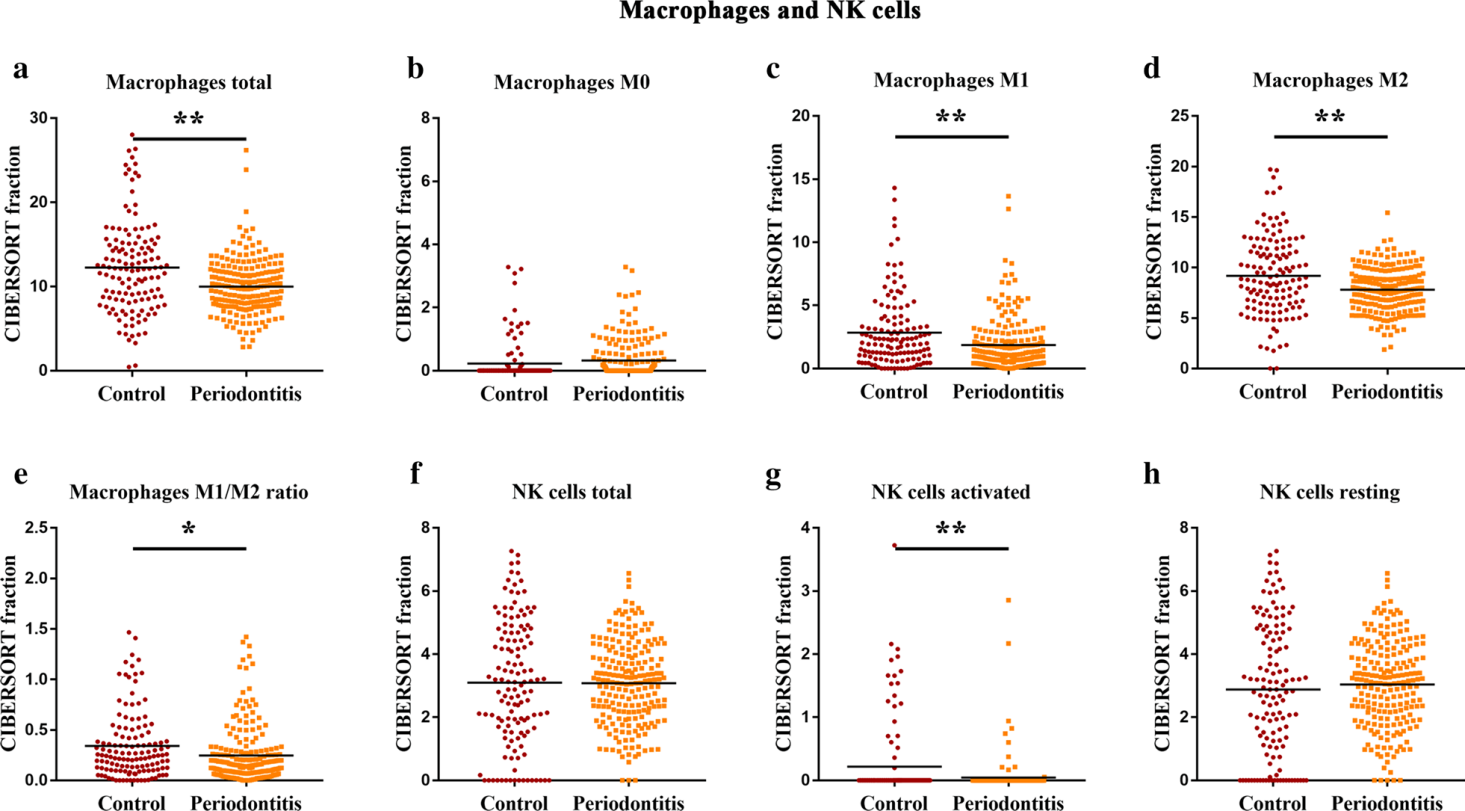
The characteristics of the differential macrophage and NK cell subpopulations between human healthy and periodontitis tissues. The CIBERSORT immune cell fractions were determined for each patient, and each dot represents a patient. The mean values and standard deviations for each cell subtype of each patient were identified for total macrophages (**a**), M0 macrophages (**b**), M1 macrophages (**c**), M2 macrophages (**d**), M1/M2 ratio (**e**), total NK cells (**f**), resting NK cells (**g**), and activated NK cells (**h**). *P < 0.05 and **P < 0.01, using Student’s *t*-test

**Fig. 5:**
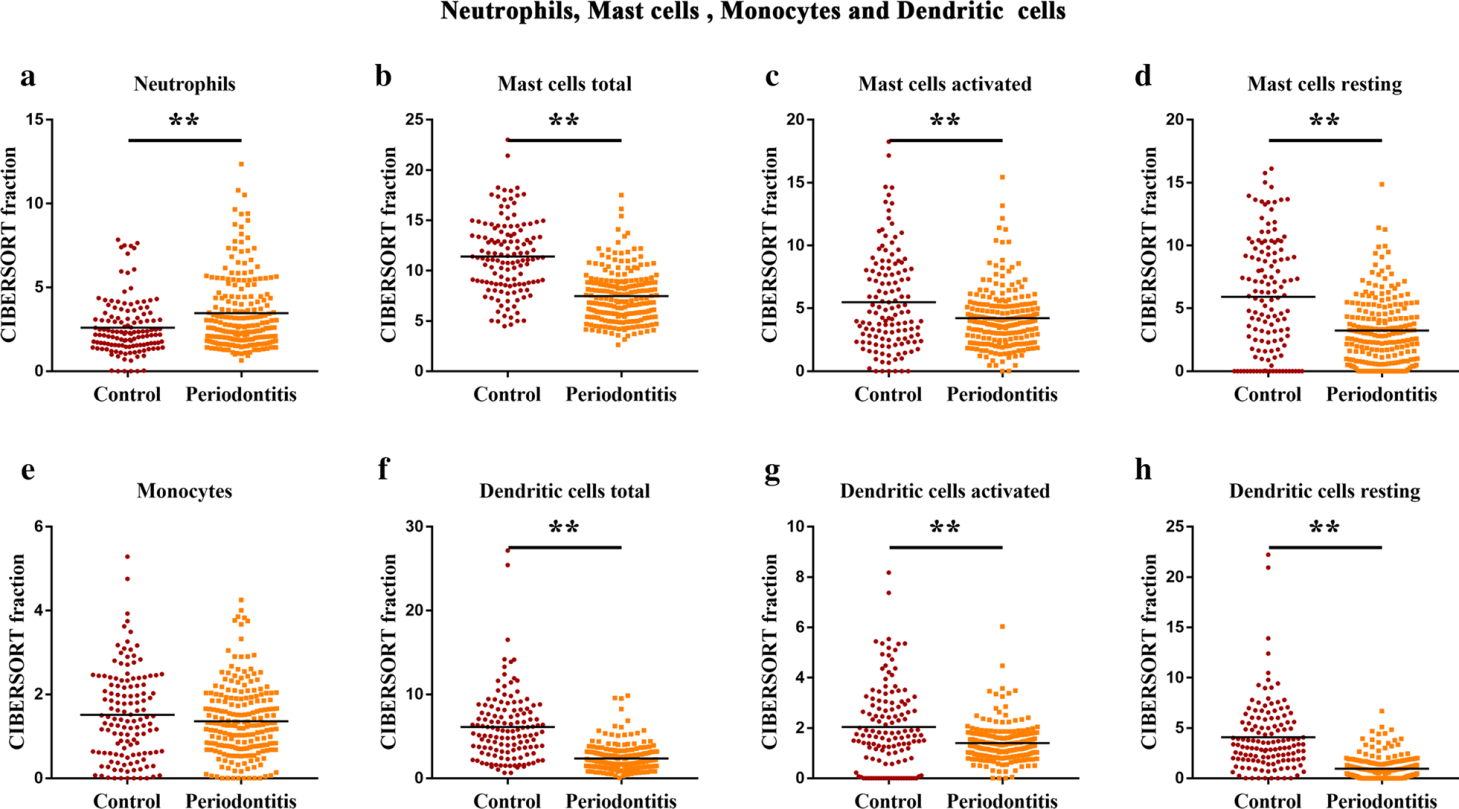
The characteristics of the differential innate immune response cells and their subtypes between human healthy and periodontitis tissues. The CIBERSORT immune cell fractions were determined for each patient, and each dot represents a patient. The mean values and standard deviations for each cell subset in each patient group were identified for neutrophils (**a**), total mast cells (**b**), the activated mast cells (**c**), the resting mast cells (**d**), monocytes (**e**), total dendritic cells (**f**), activated dendritic cells (**g**), and resting dendritic cells (**h**). *P < 0.05 and **P < 0.01, using Student’s *t*-test

Adaptive immune cells were initially assessed in chronic periodontitis, and the present data revealed that the plasma cell fraction was higher in periodontitis tissues, when compared to that in healthy periodontal tissues (Fig. 2a), while memory B cells were mainly present in healthy periodontal tissues, but less frequent in periodontitis tissues (Fig. 2d). Furthermore, naive B cells were barely visible in healthy periodontal tissues (Fig. 2c), and most of the T cells that infiltrated the healthy gums were resting memory T cells, while periodontitis tissues generally contained a higher proportion of activated CD4 memory T cells. However, the other subtypes of T cells, including resting CD4 memory T cells, CD8 T cells, follicular helper T cells and regulatory T cells (Tregs), were relatively lower in periodontitis tissues, when compared to those in healthy tissues (Fig. 3). Furthermore, it was found that although the infiltration of these immune cells was higher, when compared to healthy control tissues, the proportion of different cell subtypes varied, and the ratio of dendritic and mast cells, including both the resting and activated ones, and macrophages in periodontitis tissues was lower *vs.* healthy tissues (Figs. 4 and 5). Neutrophils were the only innate immune cells that increased in the inflamed gingival tissues (Figs. 4 and 5).

### Correlation matrix of all 22 immune cell subtypes in periodontitis tissues

**Fig. 6:**
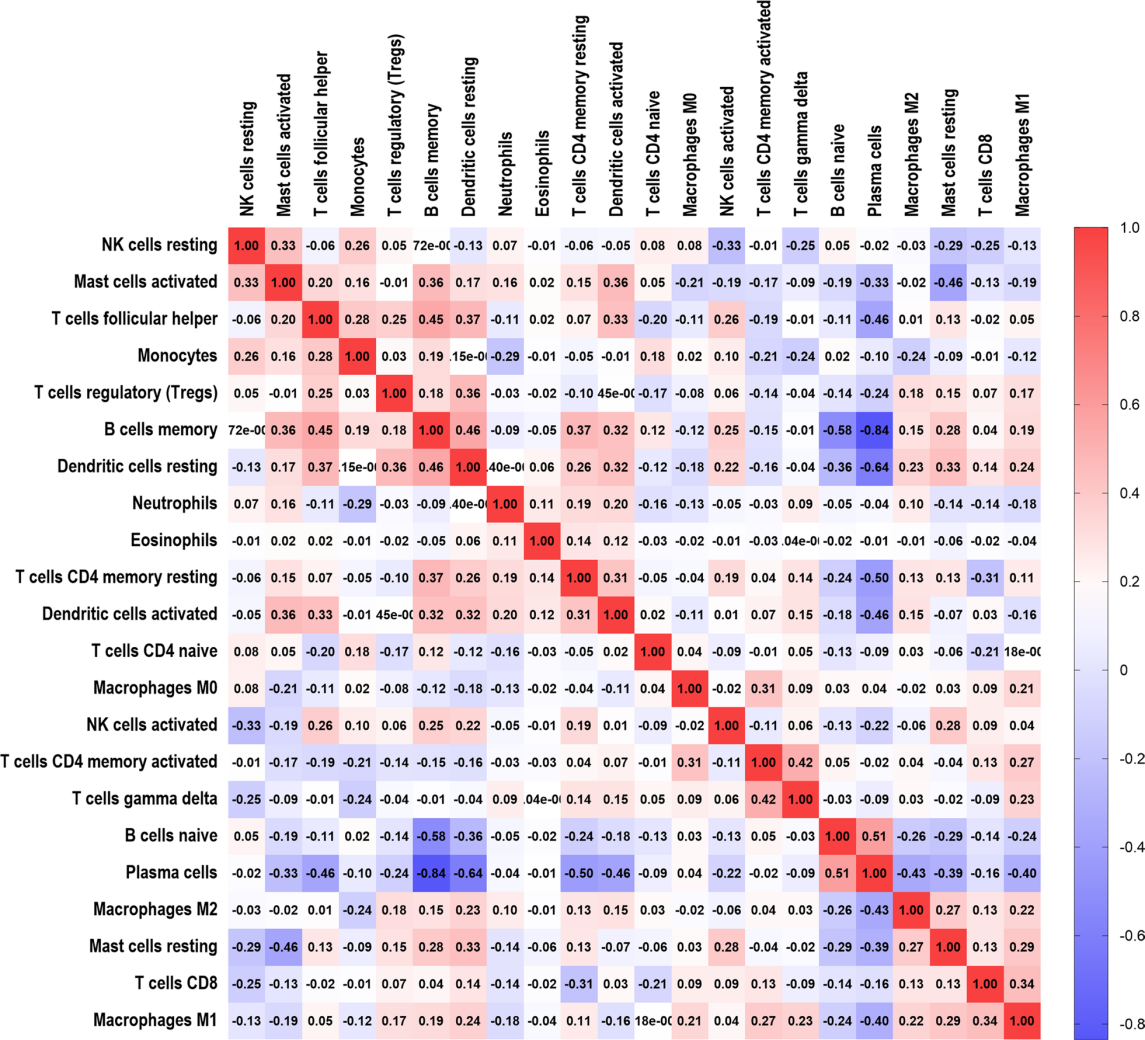
The correlation matrix of all 22 immune cell proportions. The data shows that some immune cells were negatively related (blue color), while the others were positively associated (red color). The darker the color was, the higher the correlation became (*P* < 0.05)

It was found that there was a significant inverse association between plasma cells and most of the other immune cells, that is, plasma cells versus memory B cells (γ = − 0.84), plasma cells versus resting dendritic cells (γ = − 0.64), plasma cells versus resting CD4 memory T cells (γ = 0.50), plasma cells versus activated dendritic cells (γ = − 0.46), plasma cells *vs.* T_FH_ (γ = − 0.46), plasma cells versus macrophage M2 cells (γ = − 0.43), or plasma cells *vs.* macrophage M1 cells (γ = − 0.40), in periodontitis tissues (Fig. 6). These data indicate that plasma cells might play a central role in the regulation of host immunity against periodontitis. It is possible that plasma cells replace dendritic cells, especially resting dendritic cells, and play a certain role in antigen presentation during periodontitis.

## Discussion

In the present study, the investigators profiled and identified different subtypes of infiltrating immune cells between chronic periodontitis and healthy periodontal tissues. The present data revealed that the plasma and naive B cells and neutrophils were elevated in periodontitis tissues, when compared to those in the healthy control group, while memory B cells, resting dendritic, mast and CD4 memory cells, as well as activated mast cells, M1 and M2 macrophages, and follicular helper T cells, were mainly present in healthy periodontal tissues. Furthermore, periodontitis tissues contained a higher proportion of activated CD4 memory T cells, but the other subtypes of T cells, including resting CD4 memory T cells, CD8 T cells, follicular helper T cells, and regulatory T cells (Tregs), were relatively lower in periodontitis *vs.* healthy tissues. The ratio of dendritic and mast cells and macrophages was lower in periodontitis tissues, when compared to healthy tissues. This led to the significant negative association of plasma cells with memory B cells, resting dendritic cells, resting CD4 memory T cells, activated dendritic cells, M1 macrophage, M2 macrophage, or T_FH_ between healthy controls and periodontitis tissues. In conclusion, the present present data demonstrates that different immune cells play different roles in periodontitis. Specifically, plasma cells might play a central role in the regulation of host immunity against periodontitis, while dendritic cells play a certain role in antigen presentation for the host immunity against periodontitis.

It has been well documented that immune cells possess both inflammation-promoting and -inhibiting roles in tissues. Consistently, it was found that periodontitis tissues have a marked infiltration of different immune cell subtypes, which may be significantly associated with disease development and progression [21, 22]. For example, in parallel to the increase in bacterial exposure and inflammation from healthy to periodontitis, the immune system constantly patrols and provides the surveillance of the gingival environment, and plays a pivotal role in the mediation of local immunity and maintenance of tissue homeostasis [23]. An early previous study assessed the histologic change in the gingiva during six months of abolished oral hygiene to investigate the chronic gingivitis development. The data revealed a decrease in fibroblasts, and an increase in plasma cells, while other cell types, such as lymphocytes and macrophages, remained stable, indicating the host immune responses to gingivitis development [12]. Furthermore, in order to identify these various subtypes of immune cells in tissues, different methodologies, such as immunohistochemistry and flow cytometry, which use specific antibodies, have been used, as reported in a literature [22]. However, these techniques might maximally utilize two antibodies to subtype the immune cells, which is surely not sufficient [13]. Nevertheless, gene profiles and the CIBERSORT gene signature could have more advantages in profiling and identifying the subtypes of immune cells in tissue specimens [13–15]. Thus, the present study profiled immune cell subtypes between healthy controls and periodontitis tissues using a novel CIBERSORT technology, which was previously published in the field of cancer research [14, 24]. It was observed that plasma, and B and T cells were the main immune cells in healthy periodontal tissues, while plasma cells were the major immune cells in periodontal tissues obtained from periodontitis patients, but the proportion of other types of immune cells was reduced.

A previous study revealed that the B cell lineage was the predominant cell type in periodontitis, while for plasma cells, the effector cells differentiated from B cells, which accounted for approximately 50–60% of the total infiltrating immune cells in periodontitis tissues [25]. The present data surly further confirms this previous study [25]. Indeed, previous studies have reported the production of a disease-specific antibody in the 1980s, which were conducted by Czerkinsky’s group [26–28] and Holt’s group*.*[29, 30]. Plasma cells have the ability and capacity to produce and release antibodies to conquer the microorganisms in tissues [31], that is, when bacteria invade into the connective tissues of the gum, B cells are activated and become plasma cells for antibody production, and the latter functions as the humoral immunity against periodontal bacteria [32]. Nevertheless, a recent study also demonstrated that plasma cells might also possess the ability to present antigens to B cells, which regulate the acquired humoral immunity through the negative feedback of T_FH_ [33]. The present data also confirms this negative feedback, and shows the inverse association of plasma cell level with a variety of immune cells, suggesting that these may have specific immunomodulatory effects in periodontitis development and progression. Furthermore, plasma cells play a key role in immune regulation for the secretion of various cytokines, such as interleukin-10 (IL-10), IL-35, IL-37, granulocyte–macrophage colony-stimulating factor (GM-CSF) and inducible nitric oxide synthases (iNOS), in various autoimmune and/or infectious diseases [34, 35]. Certain B cells can suppress antimicrobial immunity by producing IL-35 [34], and compared to healthy tissues, the levels of IL-35 and IL-37 were significantly elevated in gingival tissues of chronic periodontitis, indicating that infiltrating plasma cells potentially participated in and regulated the bone loss through IL-35 and IL-37 in periodontitis [36]. Another previous study revealed that plasma cell-produced IL-10 and IL-35 were able to suppress the immunity by acting on myeloid cells and T lymphocytes [37]. In the present study, it was found that the percentage of naive B cells increased, while memory B cells decreased in periodontitis. The functions of memory B cells that reside in human non-lymphoid tissues remain to be defined, and a previous study was the first to report the presence of memory B cells in healthy gingival tissues [38]. The present study also revealed the highest proportion of memory B cells in healthy control tissues, when compared to other types of immune cells. Furthermore, in periodontitis, the percentage of memory B cells decreased, and the secreting B cells were higher, when compared to the other subgroups, suggesting their participation in host humoral immunity. Although a previous study revealed the limited involvement of naive B cells in periodontal immune regulation [39], related literatures are relatively rare. The present data revealed that the proportion of naive B cells in periodontitis tissues is elevated, and the role and functions of naive B cells in periodontitis remains unclear. Thus, further studies are needed to understand the involvement in periodontal immune regulation in periodontitis.

Furthermore, the ratio of T and B cells also differ among healthy, chronic gingivitis, and adult periodontitis [25]. In innate immunity, neutrophils and various antigen presenting cells would coordinate for the preparation and action of local immunity, and during the course of the disease, the number of infiltrating immune cells would increase [21], but the proportion may differ. For example, neutrophils, as terminally differentiated leukocytes, and the key immune cells for microbial monitoring and innate responses, could accumulate in the acute phase of the disease, playing an important role in the resolution of the inflammation through the release of anti-inflammatory molecules and the organization of phagocytes [23]. In the present study, it was found that neutrophils were elevated, while macrophages and dendritic and mast cells decreased in periodontitis tissues, when compared to healthy control tissues. Furthermore, it was found that there was a high level of M2 macrophages in healthy control tissues. Functionally, macrophages can be divided into M1 and M2 macrophages [40]. M1 macrophages are able to promote the expression of inflammatory factors and bone resorption, while M2 macrophages induce tissue regeneration and repair, such as bone formation, except for the inhibition of inflammation, through the production of anti-inflammatory cytokines [41]. The present data further supports this notion.

However, the present analysis utilized the online database data, and the database lacked some particular clinical information, making it impossible for the investigators to perform the corresponding analysis, such as the analysis of each site severity. In addition, the present study was retrospective study. In order to confirm the present data, future studies with a prospective design are needed.

## Conclusion

The present study profiled and analyzed the subtypes of immune cells between healthy periodontal and chronic periodontitis tissues using online-datasets, which is just a proof-of-principle to identify the high levels of plasma and dendritic cells in periodontitis tissues. This further confirmed that these subtypes of cells could play a central role in the regulation of host immunity against periodontitis. However, further investigations are needed to understand the subtypes of immune cells in the regulation of periodontitis development and progression.

## Data Availability

The detailed data supporting the present study can be obtained upon reasonable request.

## Abbreviations

BoP: Bleeding on probing
CAL: Clinical attachment loss
GEO: Gene Expression Omnibus
PPD: Probing pocket depth
RMSE: Root mean squared error
T_FH_: Follicular B helper T cells
Tregs: Regulatory T cells
